# Hypothermia, Hallucinations, and Atrial Fibrillation Secondary to Diffuse Large B-cell Lymphoma

**DOI:** 10.7759/cureus.75560

**Published:** 2024-12-11

**Authors:** Taner B Celebi, Ahmad Shamulzai, Joe Muraca, Matthew Rice, Lisa Santoriello

**Affiliations:** 1 Family Medicine, Northwell Health, Plainview, USA; 2 Neurology, Northwell Health, Manhasset, USA; 3 Internal Medicine, University of Rochester Medical Center, Rochester, USA; 4 Emergency Medicine, Northwell Health, Manhasset, USA

**Keywords:** atrial fib, auditory hallucination, b cell neoplasm, primary lymphoma, visual hallucination

## Abstract

Diffuse large B-cell lymphoma (DLBCL) is the most common type of non-Hodgkin lymphoma (NHL) in adults, constituting a significant portion of global incidence rates. DLBCL can be further classified via genetic expression profiling into molecular subsets consisting of not-otherwise specified (NOS) subset being the most prevalent, germinal center B-cell-like (GCB) subset, and activated B-cell-like (ABC) subset. The ABC subset, marked by abnormal NF-κB signaling, is associated with poorer outcomes. This report presents an unusual case of a 30-year-old male with no past medical history who was found to have advanced-stage ABC-type DLBCL-NOS, featuring rare symptoms such as hypothermia, autonomic dysfunction, and atrial fibrillation, illustrating the unpredictable clinical manifestations of this aggressive lymphoma and the importance of molecular subtyping in treatment and prognosis.

## Introduction

Diffuse large B-cell lymphoma (DLBCL) is the most frequently diagnosed type of non-Hodgkin lymphoma (NHL) in adults, representing nearly one-third of all NHL cases in Western countries [1]. Globally, the incidence of DLBCL varies, with rates estimated between 2.3 and 13.8 cases per 100,000 person-years, while in the United States, the incidence is approximately 5.5 cases per 100,000 person-years [2-5]. Among the various subtypes of DLBCL, the most common is DLBCL, not otherwise specified (DLBCL, NOS) [6]. Gene-expression profiling has identified distinct molecular subgroups within DLBCL and NOS based on the cell of origin, namely, germinal center B-cell-like (GCB), activated B-cell-like (ABC), and unclassified [7,8]. The ABC subtype is associated with aberrant activation of NF-κB-mediated signaling, which enhances cell proliferation and inhibits pro-apoptotic mechanisms [8-10].

These molecular characteristics bear clinical significance, as the ABC subtype is linked to poorer clinical outcomes following chemotherapy compared to the GCB subtype [11]. This highlights the importance of molecular subtyping in guiding treatment strategies and assessing prognosis. In this report, we discuss a rare and atypical presentation of a young, previously healthy patient diagnosed with advanced-stage DLBCL, NOS of the ABC subtype. The patient exhibited unusual symptoms, including hypothermia, autonomic dysfunction, and atrial fibrillation, underscoring the diverse and sometimes unpredictable manifestations of this aggressive lymphoma.

## Case presentation

A 30-year-old male was found partially unresponsive in the back of his SUV during winter by a family member and brought to the hospital by Emergency Medical Services (EMS). Upon arrival, he exhibited full-body tremors and teeth clenching. His core body temperature was critically low at 81 °F, and his blood pressure was dangerously low at 76/44 mmHg. It appeared that the patient had removed his clothing and was sweating profusely in the vehicle. 

On physical examination, the patient was unresponsive, drenched in sweat, with left lateral gaze deviation, irregularly irregular pulse, and poor respiratory effort. He was promptly intubated and placed on a heating blanket, with warm fluids and vasopressors were started. 

Broad-spectrum antivirals, antifungals, and antibiotics were administered. The family provided no significant medical history, and details regarding the patient's social history were unclear for drug, alcohol, sexual history, and living conditions. The patient was admitted to the intensive care unit for further investigation and management.

**Figure 1:**
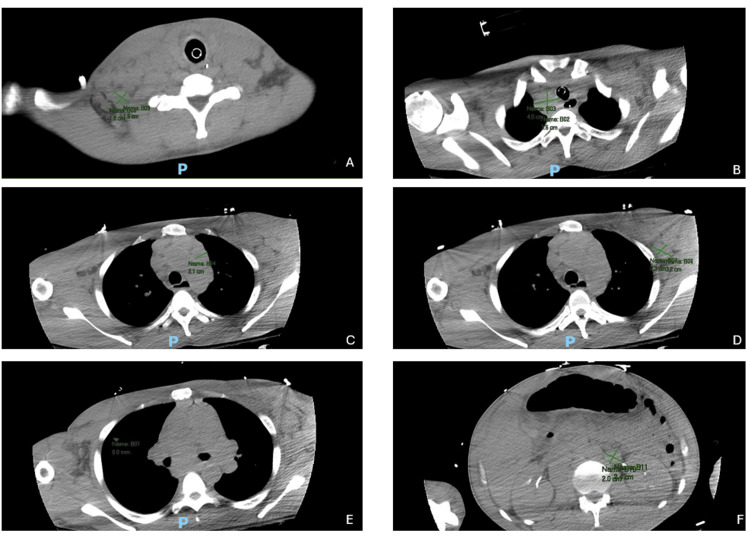
Non-contrast CT of the chest, abdomen, and pelvis showing enlarged lymph nodes. Extensive adenopathy is illustrated in the (A) right subclavicular node (1.8 cm x 1.9 cm), (B) upper right paratracheal node (4.0 cm x 3.6 cm), (C) anterior mediastinal node (2.1 cm on the short axis), (D) left axillary node (3.2 cm x 2.3 cm), (E) subcarinal node (2.9 cm on the short axis), and (F) left periaortic node (2.4 cm x 2.0 cm), with marked annotations.

With little background information, a broad approach to the workup on the patient was initiated. Significant findings were found in multiple imaging modalities. A chest X-ray revealed paratracheal mediastinal lymphadenopathy, more pronounced on the right side, along with a left hilar mass. The CT scan of the neck and chest (Figure 1) showed superior mediastinal and right paratracheal conglomerate lymphadenopathy, along with extensive adenopathy in the mediastinum, left axilla, bilateral supraclavicular regions, and retroperitoneum. In the lungs, it illustrated a small right pleural effusion with underlying passive atelectasis and an indeterminate 8 mm nodule in the right upper lobe.

**Figure 2:**
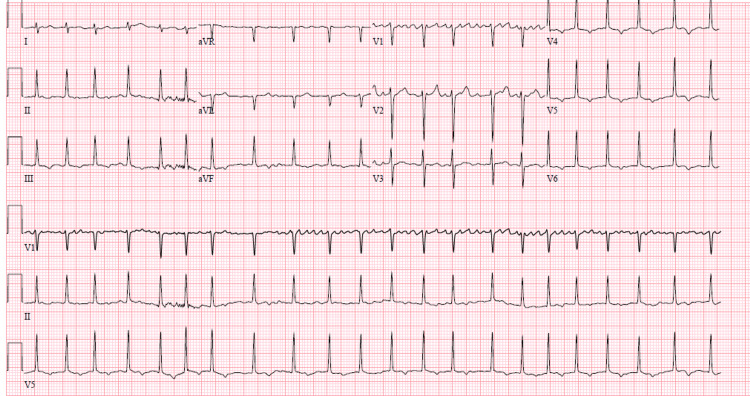
Electrocardiogram of the patient.

An EKG showing atrial fibrillation with rapid ventricular rate at 142 BPM, normal axis, QTc of 623 ms, QRS duration of 100 ms (Figure 2). A transthoracic ultrasound illustrated left ventricular systolic dysfunction with an ejection fraction of 38% and global left ventricular hypokinesis. 

While intubated, the patient was on pressor support with a Levophed drip to stabilize blood pressure for 24 hours. Empiric antibiotics covering for meningitis were administered, and a gastrointestinal (GI) polymerase chain reaction (PCR) test revealed Giardia lamblia, for which Flagyl was initiated. 

Interestingly, on admission, the patient's WBC, RBC, electrolytes, and kidney and liver function were all within normal limits. The only elevated lab work was a lactate dehydrogenase value of 878 (50-242) U/L and a minor bump in lactate at 2.1 (0.7-2.0) mmol/L. 

After three days of treatment, the patient was successfully extubated and became alert and oriented. The patient spontaneously converted from atrial fibrillation to normal sinus rhythm and began to participate in a verbal interview. During the interview, the patient denied any prior medical or surgical history, stating that he had always considered himself healthy. However, he could not fully recall the events leading up to his hospitalization.

The patient stated that he had auditory and visual hallucinations before entering his vehicle after work. He was unable to remember anything further after that point. Despite ongoing hallucinations, the patient showed signs of stabilization over two weeks, and follow-up tests were conducted as broad-spectrum treatment continued. 

The visual and auditory hallucinations were managed with Seroquel 25 mg PO. Keppra was initiated for possible seizures but later discontinued as no seizure activity was confirmed. The patient developed atrial fibrillation, initially controlled with an intravenous (IV) Cardizem drip. However, oral rate-control medications proved insufficient, necessitating amiodarone and a Digoxin load. Eventually, the patient was stabilized on Lopressor PO, and other medications for atrial fibrillation were discontinued. 

Lumbar puncture illustrated an increase in neutrophil percentage at 18% and no growth in the cultures. Flow cytometry was completed on the cerebrospinal fluid (CSF) and illustrated decreased absolute CD3, CD4, CD8, and CD19 counts, with a marked increase in CD25 interleukin 2 receptor at 39479.7 (175.3-858.2) pg/mL. Blood cultures were also found to be negative.

Ultimately, a lymph node biopsy by ENT was conducted, and the patient was diagnosed with Stage IVB Diffuse large B-cell lymphoma and began chemotherapy treatment. Although the patient was initially set to begin R-CHOP (Rituximab, Cyclophosphamide, Doxorubicin, Vincristine, and Prednisone) therapy, insurance denied coverage for the Polatuzumab component, so the patient was treated with the standard R-CHOP regimen. A right brachial peripherally inserted central catheter (PICC) line was inserted, and a left iliac bone marrow biopsy was obtained. Before chemotherapy, the patient was started on Allopurinol and Decadron for symptom control, but *B* symptoms worsened. During chemotherapy, the patient had an infusion reaction to Rituximab, managed with IV Decadron and Benadryl. Cyclophosphamide, Doxorubicin, Vincristine, Prednisone, and Rasburicase were administered, and Zarxio was started for neutropenic fever prophylaxis, later held due to a sacral decubitus ulcer infection treated with Cefepime. Granix was administered once after Zarxio was discontinued. The patient was also treated with anti-nausea medications during chemotherapy. Follow-up continued for two months post-discharge with ongoing chemotherapy and radiation treatment.

## Discussion

Many factors determine the prognosis for DLCBL. One of which is the International Prognostic Index (IPI) tool, which develops pretreatment features that predict survival in patients with aggressive NHL who will be treated with Doxorubicin chemotherapy regimens. In this metric, poorer outcomes are associated with those >60 years of age, elevated lactate dehydrogenase (LDH) levels, poor Eastern Cooperative Oncology Group (ECOG) performance status, Stage III or IV disease, and extranodal site involvement. Our patient’s International Prognostic Index (IPI) score was 3, which places him in the high-intermediate risk group, with an overall survival chance of 49% and 57% of progression-free survival [12-13]. Other factors associated with poor prognosis include elevated LDH, B2-Microglobulin, and IL-2 receptor elevations, all of which the patient displayed [13]. Additionally, this patient had the DLBCL, NOS of the ABC subtype, which is associated with poorer clinical outcomes after chemotherapy. 

Staging is important because it guides both treatment and prognosis. Our patient was diagnosed with Stage IVB, indicating widespread involvement of multiple organs or tissues beyond lymph node regions, such as the liver, lungs, or bone marrow. The staging also involves a letter, A or B, based on whether fever, weight loss, or night sweats are present. A *B* designation indicates that these symptoms are present, which is consistent with our patient's presentation [14]. 

Additionally, the ECOG score helps determine a patient’s functional status and ability to tolerate chemotherapy. The scale is measured from 0 to 5 points, with higher scores associated with lower ability to tolerate chemotherapy. Our patient’s ECOG score was 3, meaning the patient is only capable of limited self-care and confined to bed or chair for >50% of the day. However, due to his young age and high functional status before hospitalization, his ECOG score may improve over time [15]. 

The standard of care for treating DLBCL has been R-CHOP every 21 days for six cycles. However, a newer regimen, R-pola-CHP (Rituximab, Polatuzumab vedotin, Cyclophosphamide, Doxorubicin, Prednisone), which replaces Doxorubicin with Polatuzumab vedotin has shown to be effective in clinical trials. In the Phase 3 POLARIX trial, R-pola-CHP was associated with similar overall survival compared to R-CHOP. However, the 77% two-year progression-free survival and two-year event-free survival were superior to R-CHOP. Our patient was treated with R-CHOP due to insurance issues covering R-pola-CHP.

## Conclusions

This case highlights the unique unpredictable clinical presentation of atypical symptoms in a previously healthy 30-year-old male, ultimately diagnosed with Stage IVB DLBCL-NOS of the aggressive ABC subtype. The patient presented with hypothermia, atrial fibrillation, and hypotension, requiring a critical level of care. He was intubated and treated for hypothermia, and broad-spectrum antivirals, antifungals, and antibiotics were administered for empiric coverage, given the severity of his clinical presentation. Initial imaging and tests revealed extensive lymphadenopathy and elevated LDH, leading to his diagnosis following biopsy. The prognosis for DLBCL is influenced by multiple factors, including age, elevated LDH levels, ECOG performance status, and disease staging. Our patient presented with poor prognostic indicators, including an IPI score of 3 and elevated biomarkers, placing him in the high-intermediate risk group. While his ECOG score indicated limited functionality, his young age may improve his prognosis over time. Standard R-CHOP chemotherapy was initiated, with a modification to a newer regimen of R-pola-CHP, which has shown better outcomes in clinical trials. He experienced complications like an infusion reaction and neutropenia, but with ongoing chemotherapy and radiation, the patient showed signs of recovery, and follow-up care continued for two months post-discharge. This case conveys the importance of staging and molecular profiling in treating DLBCL while elucidating this fascinating patient presentation to aid clinicians in understanding the vast array of potential DLBCL clinical manifestations.
